# Regulation of ADAMTS-1, -4 and -5 expression in human macrophages: Differential regulation by key cytokines implicated in atherosclerosis and novel synergism between TL1A and IL-17^☆^

**DOI:** 10.1016/j.cyto.2013.06.315

**Published:** 2013-10

**Authors:** Tim G. Ashlin, Alvin P.L. Kwan, Dipak P. Ramji

**Affiliations:** Cardiff School of Biosciences, Cardiff University, Sir Martin Evans Building, Museum Avenue, Cardiff CF10 3AX, United Kingdom

**Keywords:** ADAMTS, a disintegrin and metalloproteinase with thrombospondin motifs, ApoB, apolipoprotein B, ApoE, apolipoprotein E, DR3, death receptor 3, ECM, extracellular matrix, GAPDH, glyceraldehyde-3-phosphate dehydrogenase, HMDM, human monocyte-derived macrophages, IFN-γ, interferon-γ, IL, interleukin, LDL, low-density lipoprotein, LDLR, LDL receptor, LPL, lipoprotein lipase, MMP, matrix metalloproteinase, PMA, phorbol 12-myristate 13-acetate, RT-qPCR, real-time quantitative polymerase chain reaction, TGF-β, transforming growth factor-β, TL1A, tumour necrosis factor-like protein 1A, TNF-α, tumour necrosis factor-α, VSMC, vascular smooth muscle cells, ADAMTS proteases, Atherosclerosis, Cytokines, Macrophages, Gene expression

## Abstract

Atherosclerosis is an inflammatory disease of the vasculature regulated by cytokines. Macrophages play a crucial role at all stages of this disease, including regulation of foam cell formation, the inflammatory response and stability of atherosclerotic plaques. For example, matrix metalloproteinases produced by macrophages play an important role in modulating plaque stability. More recently, the ADAMTS proteases, which are known to play a key role in the control of cartilage degradation during arthritis, have been found to be expressed in atherosclerotic lesions and suggested to have potentially important functions in the control of plaque stability. Unfortunately, the action of cytokines on the expression of ADAMTS family in macrophages is poorly understood. We have investigated the effect of classical cytokines (IFN-γ and TGF-β) and those that have been recently identified (TL1A and IL-17) on the expression of ADAMTS-1, -4 and -5 in human macrophages. The expression of all three ADAMTS members was induced during differentiation of monocytes into macrophages. TGF-β had a differential action with induction of ADAMTS-1 and -5 expression and attenuation in the levels of ADAMTS-4. In contrast, IFN-γ suppressed the expression of ADAMTS-1 without having an effect on ADAMTS-4 and -5. Although TL-1A or IL-17A alone had little effect on the expression of all the members, they induced their expression synergistically when present together. These studies provide new insight into the regulation of key ADAMTS family members in human macrophages by major cytokines in relation to atherosclerosis.

## Highlights

• Atherosclerosis is an inflammatory disorder regulated by cytokines.
• ADAMTS proteases have been suggested to play an important role in this disease.
• The action of key cytokines on the expression of ADAMTS proteases in macrophages is poorly understood.
• The effect of IFN-γ, TGF-β, TL1A and IL-17A on the expression of ADAMTS-1, -4 and -5 was studied.
• Novel differential actions and synergistic interactions were identified.

## 1 Introduction

Atherosclerosis is a progressive disease characterised by lipid accumulation and inflammation within the walls of the large and medium arteries [1]. The disease is initiated by the activation of the arterial endothelium by a range of risk factors leading to infiltration of immune cells, particularly T-lymphocytes and monocytes [1]. The latter then differentiate into macrophages and take up modified low-density lipoproteins (LDL), particularly oxidized LDL, to form lipid laden foam cells that characterise the fatty streak seen in the early stages of the disease. As the disease progresses, complex lesions develop that are usually covered with a fibrous cap composed of vascular smooth muscle cells (VSMCs) and extracellular matrix (ECM) molecules. The fibrous cap encloses a lipid-rich necrotic core consisting of modified LDL, cholesterol and apoptotic/necrotic cells. The acute symptoms of atherosclerosis usually do not occur due to the plaque critically narrowing the artery but when an unstable plaque ruptures leading to a thrombotic reaction [2]. The stability of a mature atherosclerotic plaque, which is dictated by a balance between ECM synthesis and degradation, is therefore very important in controlling acute events such as heart attack and stroke [3].

ADAMTS proteases are a family of proteins that share a similar domain pattern and substrate range; they are structurally related to the matrix metalloproteinase (MMP) family and have been implicated in a number of pathophysiological conditions including osteoarthritis and, more recently, atherosclerosis [4–6]. The founding member of the ADAMTS proteases was first cloned, identified and named in a study carried out in 1997 [7], from here the family has grown to 19 members [4,5]. ADAMTS proteases are secreted enzymes that act on a wide variety of ECM substrates including pro-collagen, proteoglycans, hyalectans and cartilage oligomeric matrix protein [4,8].

MMPs have been suggested to be major regulators of the atherosclerotic process through re-modeling of the plaque ECM [9]. As the protein families are structurally related, the role for ADAMTS proteins in atherosclerosis could be similar to that of the MMPs [4]. The central hypothesised role of the ADAMTS proteases within the atherosclerotic plaque is cleavage of versican potentially leading to regulation of migration, proliferation, apoptosis and other cellular events within VSMC and macrophages [4]. ADAMTS-1, -4, -5 and -8 are expressed within human atherosclerotic plaques, and macrophages have been identified as major contributors towards ADAMTS expression in the disease [10–12]. ADAMTS proteases are also expressed in VSMC and endothelial cells, but to a lower extent than macrophages and foam cells [10,11]. ADAMTS-1 expression has been studied in various mouse tissues and has been shown to be at the highest level in the aorta [11]. In addition, ADAMTS-4 mRNA is present in the aortas of the LDL receptor (LDLR)^−/−^ apolipoprotein B (ApoB)^100/100^ mice before any atherosclerotic lesions are visible and the level of expression increased as the lesions become more advanced [10]. In separate studies, serum levels of ADAMTS-4 have also shown a significant correlation with the severity of coronary artery disease [13,14]. More recently, ADAMTS-5 has been found to have a novel role in proteoglycan turnover and lipoprotein retention in atherosclerosis [15]. These findings, taken together, outline the potential regulatory role that ADAMTS proteases could have over the stability of the atherosclerotic plaque.

Despite the potentially important link between ADAMTS family and atherosclerosis, limited *in vitro* studies have been carried out on their regulation in macrophages by cytokines in relation to this disease. The first study investigated the expression and regulation of ADAMTS proteases in macrophages, before and after differentiation, by IFN-γ, IL-1β and tumour necrosis factor (TNF)-α [10]. The expression of ADAMTS-4 and -8 was induced upon monocyte to macrophage differentiation and macrophage expression of ADAMTS-4, -7, -8 and -9 mRNA was further enhanced upon stimulation with IFN-γ or TNF-α. On the other hand, IFN-γ attenuated the expression of ADAMTS-1 [10]. The second study analysed the effect of TGF-β stimulation on ADAMTS-4 expression in macrophages [16]. This anti-atherogenic cytokine inhibited the expression of ADAMTS-4 and small interfering RNA-mediated knockdown revealed a critical role for Smads, p38 mitogen-activated protein kinase and c-Jun in this response [16]. These findings together suggest potentially important roles for ADAMTS proteases during atherosclerosis, demonstrate how regulation by specific cytokines can influence their expression, and highlight the need for further studies aimed to identifying the effect of different cytokines implicated in this disease on the expression of ADAMTS family members.

The objective of this study was therefore to investigate the action of classical cytokines (TGF-β, IFN-γ) and those that have been more recently identified (TL1A and IL-17A) on the expression of ADAMTS-1, -4 and -5 in human macrophages. *In vivo* studies in mouse model systems have highlighted a pro-atherogenic role of IFN-γ [17,18] and an anti-atherogenic action of TGF-β [19]. The role of TL1A, which interacts with death receptor 3 (DR3), in atherosclerosis *in vivo* has not been investigated but *in vitro* studies indicate that the cytokine promotes foam cell formation [20]. In addition, in combination with IFN-γ, the TL1A/DR3 axis has been shown to have a role in atherosclerosis through stimulation of MMP-9, potentially leading to de-stabilisation of the plaque [21]. IL-17A has been regarded previously as pro-inflammatory as this cytokine has been shown to induce many mediators such as TNF-α and IL-1 [22]. However, its role in the development of atherosclerosis *in vivo* remains controversial with both pro- and anti-atherogenic actions being reported [23].

## 2 Materials and methods

### 2.1 Reagents

All chemicals were purchased from Sigma–Aldrich (Poole, UK) unless otherwise stated. Recombinant human TGF-β, IFN-γ, TL1A and IL-17A were supplied by Peprotech (London, UK).

### 2.2 Cell culture

Most experiments were carried out in the human acute leukaemia cell line (THP-1) with key finding confirmed in human monocyte-derived macrophages (HMDM). The latter were obtained by differentiation of monocytes isolated from buffy coats supplied by the Welsh Blood service using Ficoll-Hypaque purification described elsewhere [24,25]. The cells were grown in complete RPMI-1640 supplemented with 10% (v/v) heat-inactivated FCS (56 °C, 30 min), penicillin (100 U/ml), streptomycin (100 μg/ml) and l-glutamine (2 mmol/L) at 37 °C in a humidified atmosphere containing 5% (v/v) CO_2_. THP-1 monocytes were differentiated into macrophages using 160nM phorbol 12-myristate 13-acetate (PMA) for 24 h. In all experiments, unless otherwise stated, macrophages were incubated with TGF-β (30 ng/ml), IFN-γ (1000U/ml), TL1A (100 ng/ml), IL-17A (100 ng/ml) or TL1A and IL-17A for 24 h. Recombinant human TGF-β, IFN-γ, TL1A and IL-17A were reconstituted in PBS/0.1% BSA that was subsequently used as a vehicle control.

### 2.3 Real-time quantitative PCR (RT-qPCR)

RNA extraction, reverse transcription and qPCR analysis were performed as described elsewhere [24,25]. The sequences of oligonucleotides, which were purchased from Sigma Aldrich (Poole, UK), are shown in Supplementary Table I. Fold changes in expression were calculated using 2^−(Δ^*^Ct^*^1–Δ^*^Ct^*^2)^, where Δ*Ct* represents the difference between the threshold cycle (*C_T_*) for each target gene and housekeeping glyceraldehyde-3-phosphate dehydrogenase (GAPDH) mRNA transcript levels [25]. Melting curve analysis was performed on each primer set to confirm amplification of a single product and all amplicons were sequenced to ensure reaction specificity (data not shown).

### 2.4 Western blotting

Total cell lysates were size-fractionated and analysed by western blotting as previously described [24,25]. Samples were subjected to electrophoresis alongside comparative molecular weight markers (GE Healthcare, Wisconsin, USA) to determine the size of the protein product. An antibody specific to ADAMTS-4 (PA1-1749) was supplied by Thermo Fisher Scientific (Northumberland, UK). Antibodies specific to apoE (0650-1904) and β-actin (A2228) were supplied by Biogenesis (Poole, UK) and Sigma (Poole, UK) respectively.

### 2.5 Statistical analysis

All data are presented as mean (±standard deviation (SD) on the assigned number of independent experiments where, in experiments involving HMDM, this refers to the number of independent experiments performed using samples from different donors. Data sets were tested for normality using the Shapiro–Wilk test. Statistical analysis was carried out using either a Student’s *t*-test (two-tailed, paired) or one-way ANOVA with Tukey’s post hoc test, where homogeneity of variance was met; or Welch’s test of equality of means with Games–Howell post hoc analysis. Results were regarded as significant if *P* ⩽ 0.05.

## 3 Results

### 3.1 Macrophage differentiation induced the expression of ADAMTS-1, -4 and -5

**Fig. 1:**
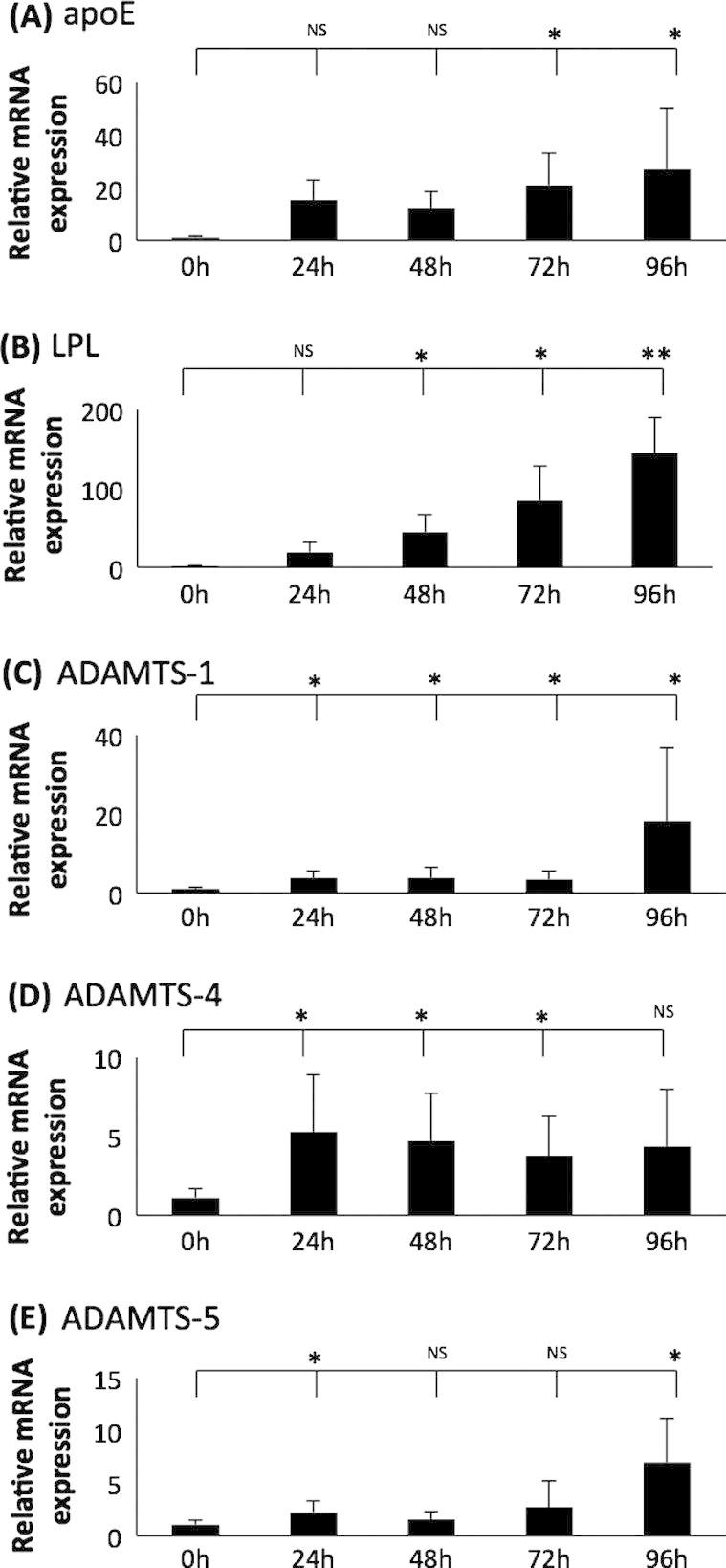
The expression of ADAMTS-1, -4 and -5 is induced during differentiation of THP-1 monocytes into macrophages. THP-1 monocytes were treated with 160 nM PMA for the indicated period of time and total cellular RNA was subjected to RT-qPCR using primers against (A) apoE, (B) LPL, (C) ADAMTS-1, (D) ADAMTS-4 and (E) ADAMTS-5. The mRNA expression levels were calculated using the comparative Ct method and normalised to GAPDH mRNA levels with cells at 0 h given an arbitrary value of 1. Data represent the mean ± SD of 4 independent experiments for apoE and LPL, and 7 independent experiments for ADAMTS-1, -4 and -5. Statistical analysis was performed using one-way ANOVA (^*^P < 0.05; ^**^P < 0.01; NS, not significant).

It was of interest to investigate how the expression of ADAMTS-1, -4 and -5 was regulated during monocyte-macrophage differentiation. These experiments were carried out on the THP-1 cell line which is widely utilised for such investigations as the responses in them are conserved with primary macrophages and *in vivo* conditions [24,26]. Indeed, this cell line has been used in previous publications to study the regulation of ADAMTS expression in human macrophages [10,16,27]. THP-1 monocytes are readily differentiated into macrophages after stimulation with PMA [26,27]. Previous studies have shown that the expression of apolipoprotein E (apoE) and lipoprotein lipase (LPL) is increased during PMA-induced differentiation of THP-1 monocytes into macrophages [28,29] and they were therefore included as positive controls. The expression of apoE and LPL mRNA was indeed significantly induced upon PMA-mediated differentiation of THP-1 cells (Fig. 1, panels A and B). Similarly, ADAMTS-1, -4 and -5 were expressed in THP-1 monocytes and their levels increased significantly during differentiation into macrophages (Fig. 1, panels C–E). Although the expression of ADAMTS-5 failed to reach significance at 48 h and 72 h, the levels were higher than those seen in monocytes.

**Fig. 2:**
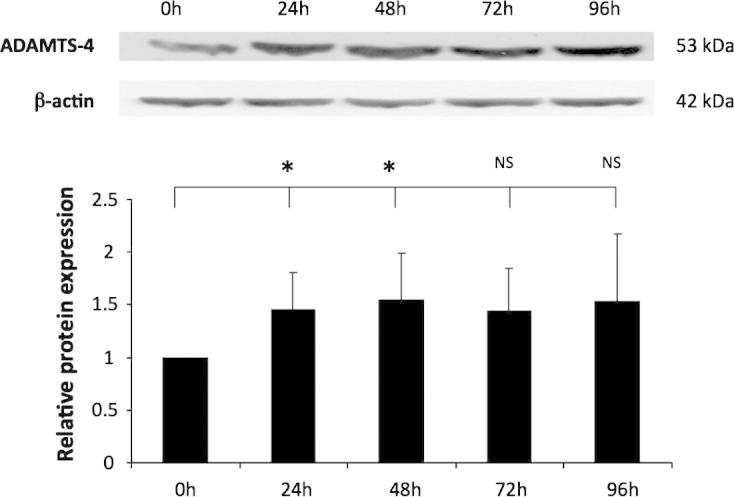
The expression of the ADAMTS-4 protein is induced during differentiation of THP-1 monocytes into macrophages. THP-1 monocytes were treated with 160 nM PMA for the indicated period of time and equal amount of total cellular protein was subjected to Western blot analysis using antisera against ADAMTS-4 or β-actin as indicated. Protein expression, as determined by densitometric analysis, was normalised to β-actin and is displayed as a fold change compared to 0 h (arbitrarily assigned as 1). Data represent the mean ± SD of three independent experiments. Statistical analysis was performed using Student’s *t* test (^*^P < 0.05; NS, not significant).

The expression of ADAMTS-4 protein was also analysed by western blot analysis. As shown in Fig. 2, the expression of ADAMTS-4 was significantly increased after 24 and 48 h of PMA stimulation. Although the levels of ADAMTS-4 at 72 h and 96 h did not reach significance, they were much higher than those in monocytes.

Overall, therefore, the induction of ADAMTS-1, -4 and -5 expression was significant after 24 h of PMA stimulation in all cases during RT-qPCR and, in the case of ADAMTS-4, by Western blot analyses. For these reasons, all subsequent experiments that investigated gene expression in THP-1 macrophages utilised a 24 h differentiation period with PMA.

### 3.2 TGF-β attenuated the expression of ADAMTS-4 and increased the expression of ADAMTS-1 and -5 in human macrophages

TGF-β is highly expressed in atherosclerotic plaques and has been implicated in several cellular changes during this disease [19]. TGF-β predominantly shows anti-atherogenic properties, highlighted by low serum levels being observed in patients with advanced atherosclerosis and regions of the aorta with a high probability of lesion development displaying low levels of TGF-β expression [19]. In addition, the inhibition of TGF-β activity and/or expression in mouse models of atherosclerosis results in accelerated lesion development and an elevated inflammatory response [19,25]. The action of TGF-β on the expression of ADAMTS-1, -4 and -5 was therefore investigated. ApoE, whose expression is induced by TGF-β, was included as a positive control.

**Fig. 3:**
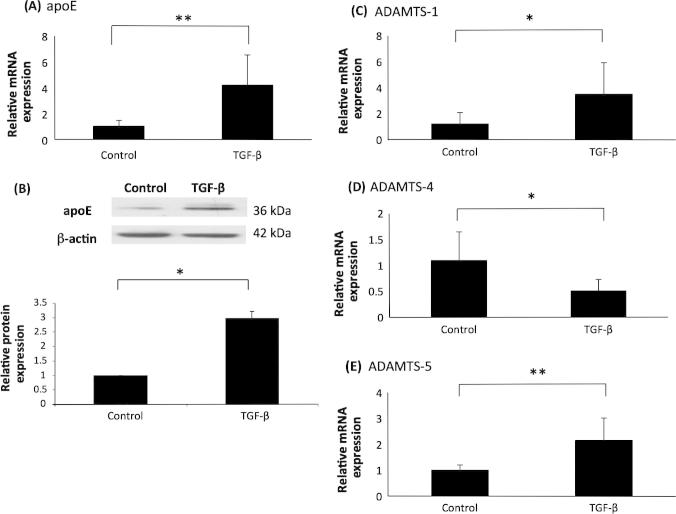
Differential action of TGF-β on the expression of ADAMTS-1, -4 and -5 in human macrophages. THP-1 macrophages were treated for 24 h with vehicle (Control) or 30 ng/ml TGF-β. (A, C, D, E), total cellular RNA was isolated and subjected to RT-qPCR using primers against (A) apoE, (C) ADAMTS-1, (D) ADAMTS-4 and (E) ADAMTS-5. The mRNA expression levels were calculated using the comparative Ct method and normalised to GAPDH mRNA levels with those from control, vehicle-treated cells given an arbitrary value of 1. Data represent the mean ± SD of 3 independent experiments for apoE and 4 independent experiments for ADAMTS-1, -4 and -5. (B), equal amounts of total cellular protein were subjected to Western blot analysis using antisera against apoE or β-actin as indicated. Protein expression as determined by densitometric analysis, was normalised to β-actin and is displayed as a fold change compared to control (arbitrarily assigned as 1). Data represent the mean ± SD of 3 independent experiments. Statistical analysis was performed using Student’s t test (^*^, *P *< 0.05; ^**^, *P *< 0.01; NS, not significant).

Consistent with previous studies [30,31], the expression of apoE mRNA and protein was induced by TGF-β (Fig. 3, panels A and B). In addition, as expected [16], the cytokine attenuated the expression of ADAMTS-4 mRNA (Fig. 3, panel D). In contrast, TGF-β induced the expression of ADAMTS-1 and -5 mRNA (Fig. 3, panels C and E).

### 3.3 IFN-γ attenuated the expression of ADAMTS-1 and had no effect on the expression of ADAMTS-4 and -5 in human macrophages

Studies *in vitro* have suggested a complex role for IFN-γ with both pro- and anti-atherogenic effects [17,18,32]. However, the evidence from *in vivo* studies is clearer, and it has been demonstrated that chronic administration of recombinant IFN-γ enhanced atherosclerosis in apoE^−/−^ mice [17]. Also, within apoE^−/−^ and LDLR^−/−^ mice, genetic ablation of IFN-γ, or the IFN-γ receptors reduced atherosclerosis [17]. The action of IFN-γ on the expression of ADAMTS-1, -4 and -5 was therefore investigated. ApoE, whose expression is inhibited by IFN-γ [33], was included as a positive control.

**Fig. 4:**
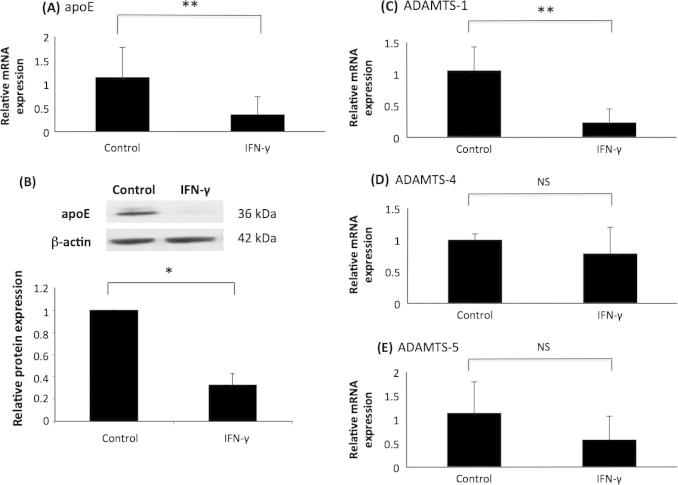
Differential action of IFN-γ on the expression of ADAMTS-1, -4 and -5 in human macrophages. THP-1 macrophages were treated for 24 h with vehicle (Control) or 1000U/ml IFN-γ. (A, C, D, E), total cellular RNA was isolated and subjected to RT-qPCR using primers against (A) apoE, (C) ADAMTS-1, (D) ADAMTS-4 and (E) ADAMTS-5. The mRNA expression levels were calculated using the comparative Ct method and normalised to GAPDH mRNA levels with those in control, vehicle-treated cells given an arbitrary value of 1. Data represent the mean ± SD of 3 independent experiments. (B), equal amount of total cellular protein was subjected to Western blot analysis using antisera against apoE or β-actin as indicated. Protein expression as determined by densitometric analysis, was normalised to β-actin and is displayed as a fold change compared to control (arbitrarily assigned as 1). Data represent the mean ± SD of 3 independent experiments. Statistical analysis was performed using Student’s *t* test (^*^P < 0.05; ^**^P < 0.01; NS, not significant).

**Fig. 5:**
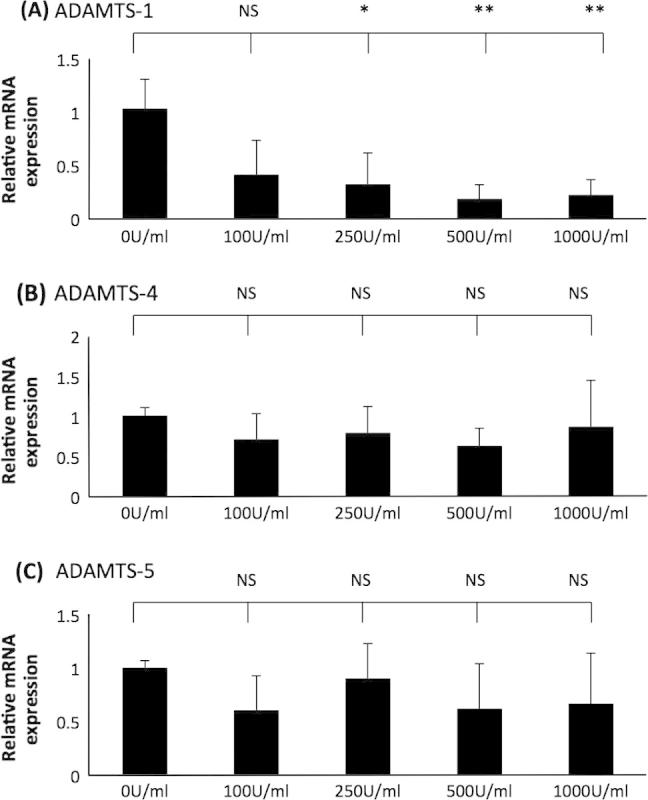
The effect of different concentrations of IFN-γ on the expression of ADAMTS-1, -4 and -5 in THP-1 macrophages. THP-1 macrophages were treated for 24 h with different concentrations of IFN-γ as indicated. Total cellular RNA was then isolated and subjected to RT-qPCR using primers against (A) ADAMTS-1, (B) ADAMTS-4 and (C) ADAMTS-5. The mRNA expression levels were calculated using the comparative Ct method and normalised to GAPDH mRNA levels with samples from cells treated with 0 U/ml of cytokine given an arbitrary value of 1. Data represent the mean ± SD of 3 independent experiments. Statistical analysis was performed using one-way ANOVA (^*^P < 0.05; ^**^P < 0.01; NS, not significant).

**Fig. 6:**
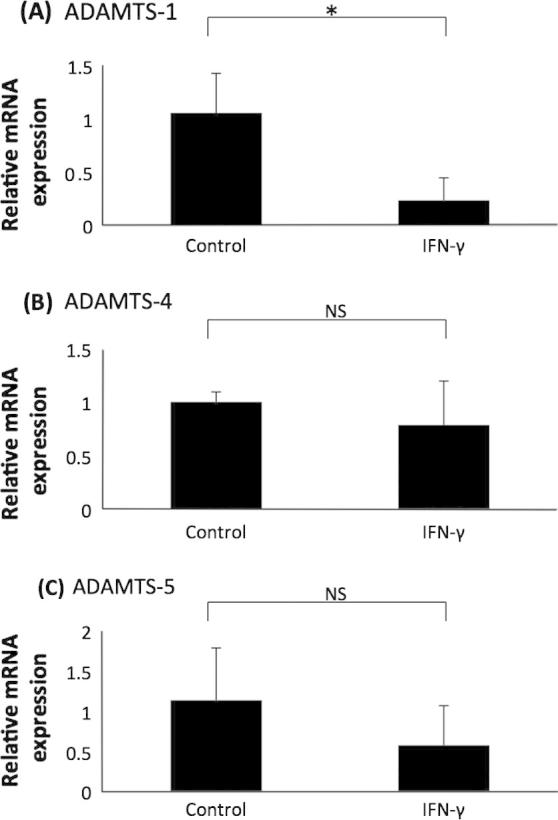
The effect of IFN-γ on the expression of ADAMTS-1, -4 and -5 in primary HMDM. HMDM were treated for 24 h with vehicle (Control) or 1000 U/ml IFN-γ. Total cellular RNA was then isolated and subjected to RT-qPCR using primers against (A) ADAMTS-1, (B) ADAMTS-4 and (C) ADAMTS-5. The mRNA expression levels were calculated using the comparative Ct method and normalised to GAPDH mRNA levels with those in control, vehicle-treated cells given an arbitrary value of 1. Data represent the mean ± SD of 3 independent experiments. Statistical analysis was performed using Student’s *t* test (^*^P < 0.05; NS, not significant).

As expected, the expression of apoE mRNA and protein was attenuated by IFN-γ (Fig. 4, panels A and B). Similarly, ADAMTS-1 mRNA expression was significantly reduced by IFN-γ stimulation (Fig. 4, panel C). In contrast, IFN-γ had no significant effect on the expression of ADAMTS-4 and -5 mRNA (Fig. 4, panel D and E). The concentration of IFN-γ used in these experiments was based on previous studies investigating the effect of this cytokine in the control of macrophage gene expression [32,34]. The results obtained here differed slightly to another previously published study that utilised 100 U/ml IFN-γ [10]. In order to investigate whether the differences were due to the concentration of the cytokine used, a dose response experiment was carried out. The studies confirmed that ADAMTS-1 mRNA expression was reduced by IFN-γ stimulation; a significant reduction in expression was observed after 250 U/ml, 500 U/ml and 1000 U/ml of the cytokine (Fig. 5, panel A). On the other hand, ADAMTS-4 and -5 expression exhibited no significant change at all concentrations of IFN-γ stimulation (Fig. 5, panels B and C). In order to further confirm that the results obtained were not peculiar to the THP-1 cell line, representative experiments were performed on primary HMDM. Similar to THP-1 macrophages, IFN-γ attenuated the expression of ADAMTS-1 and had no significant effect on the expression of ADAMTS-4 and -5 in HMDM (Fig. 6).

### 3.4 TL1A and IL-17A together, but not alone, induce the expression of ADAMTS-1, -4 and -5 in human macrophages

**Fig. 7:**
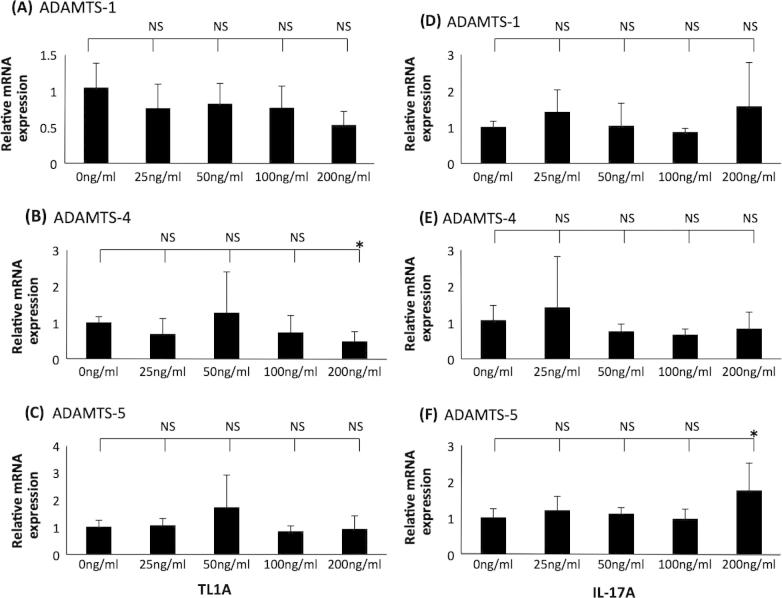
The effect of different concentrations of TL1A or IL-17 on the expression of ADAMTS-1, -4 and -5 in THP-1 macrophages. THP-1 macrophages were treated for 24 h with different concentrations of (A–C) TL1A or (D–F) IL-17 as indicated. Total cellular RNA was then isolated and subjected to RT-qPCR using primers against (A and D) ADAMTS-1, (B and E) ADAMTS-4 and (C and F) ADAMTS-5. The mRNA expression levels were calculated using comparative Ct method and normalised to GAPDH mRNA levels with samples from cells treated with 0 ng/ml of the cytokine given an arbitrary value of 1. Data represent the mean ± SD of 3 independent experiments. Statistical analysis was performed using one-way ANOVA (^*^P < 0.05; NS, not significant).

As detailed above, both TL-1A and IL-17 have been shown to have pro-atherogenic actions *in vitro* [20–22]. The action of TL1A or IL-17A on the expression of ADAMTS-1, -4 and -5 was therefore investigated. Consistent with previous studies [20], TL1A inhibited the expression of apoE mRNA in THP-1 macrophages (Supplementary Fig. 1, panel A). In contrast, no significant change was observed in the expression of ADAMTS-1, -4 and -5 (Supplementary Fig. 1, panels B–D). Similarly, no significant effect of IL-17A on the expression of ADAMTS-1, -4 and -5 were seen though, consistent with its pro-atherogenic role *in vitro*, the cytokine inhibited apoE mRNA expression (Supplementary Fig. 2). The concentration of 100 ng/ml of cytokine in these experiments was based on previous research that investigated the TL1A- or IL-17A-mediated regulation of gene expression [20,21,35]. In order to rule out the possibility that the results were because of the concentration of TL1A or IL-17A used, a dose response experiment was carried out. As shown in Fig. 7, TL1A had no significant effect on the expression of ADAMTS-1 and -5 at all concentrations employed (panels A and C). In addition, this cytokine had no significant effect on ADAMTS-4 expression at concentration of 25 ng/ml, 50 ng/ml and 100 ng/ml (Fig. 7, panel B). However, a statistically significant reduction of ADAMTS-4 expression was observed at the highest concentration of TL1A used (200 ng/ml) (Fig. 7, panel B). For IL-17A, there was no statistically significant effect on the expression of ADAMTS-1 and -4 (Fig. 7, panels D and E). Although, IL-17A also had no significant effect on ADAMTS-5 expression at 25 ng/ml, 50 ng/ml or 100 ng/ml, a statistically significant induction was seen at the highest concentration of 200 ng/ml (Fig. 7, panel F).

IL-17A has been shown to induce the production of pro-inflammatory cytokines from human macrophages [36] and there is also an increasing volume of literature suggesting that IL-17A can act synergistically with cytokines such as TNF-α, IL-22 and IFN-γ to enhance pro-inflammatory responses [37–40]. Similarly, TL1A has also been implicated in modulating pro-inflammatory responses from other cytokines: TL1A has been shown to synergise with IL-12 and IL-18 to enhance the production of IFN-γ from T-cells and NK cells [41]. The synergy between the same agents was also observed when TL1A augmented the IL-12/IL-18-induced IFN-γ production from CCR9^+^CD4^+^PB T-cells [42]. In addition, TL1A has been shown to synergise with IFN-γ to produce various pro-inflammatory responses from THP-1 macrophages [21].

**Fig. 8:**
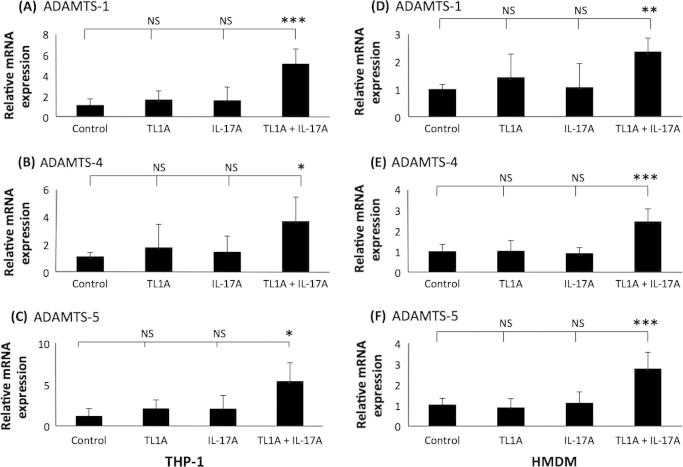
TL1A and IL-17 together induce the expression of ADAMTS-1, -4 and -5 in THP-1 macrophages and primary HMDM. (A–C) THP-1 macrophages or (D–F) HMDM were treated for 24 h with either vehicle (Control) or 100 ng/ml TL-1A and 100 ng/ml IL-17A alone or together, as indicated. Total cellular RNA was then isolated and subjected to RT-qPCR using primers against (A and D) ADAMTS-1, (B and E) ADAMTS-4 and (C and F) ADAMTS-5. The mRNA expression levels were calculated using comparative Ct method and normalised to GAPDH mRNA levels with samples from cells treated with vehicle given an arbitrary value of 1. Data represent the mean ± SD of 3 independent experiments. Statistical analysis was performed using one-way ANOVA (^*^P < 0.05; ^**^P < 0.01; ^***^P < 0.001; NS, not significant).

In the light of the findings detailed above, the effect of co-stimulation of THP-1 macrophages and HMDM with TL1A and IL-17A on the expression of ADAMTS-1, -4 and -5 was investigated. As shown in Fig. 8, as expected, TL1A or IL-17A alone failed to significantly affect the expression of all three ADAMTS members in both cellular systems. However, a statistically significant induction of ADAMTS-1, -4 and -5 expressions were seen when THP-1 macrophages and HMDMs were co-stimulated with the two cytokines (Fig. 8). In contrast, TL1A or IL-17A did not affect the action of IFN-γ on ADAMTS-1, -4 and -5 expressions (Supplementary Fig. 3).

## 4 Discussion

Recent studies have shown that ADAMTS proteases are expressed within the atherosclerotic plaque [4,12]. The action of the proteases within the plaque could potentially lead to regulation of plaque stability through various mechanisms [4]. Unfortunately, the action of key cytokines implicated in the control of inflammation during atherosclerosis on ADAMTS members is poorly understood. The current investigations aimed to increase understanding of how the expression of ADAMTS-1, -4 and -5 are regulated by cytokines in human macrophages within the atherosclerotic plaque [1,43].

The data presented in this study demonstrated that ADAMTS-1, -4 and -5 were expressed in THP-1 macrophages and this was increased significantly during monocyte-macrophage differentiation (Figs. 1 and 2). Previously, a study published in 2003 showed that THP-1 monocytes expressed ADAMTS-4 and upon PMA stimulation, the expression was significantly increased [27]. The same study also demonstrated that ADAMTS-4 expression was suppressed by anti-atherogenic peroxisome proliferator-activated receptor-γ and retinoid X receptor agonists [27]. Another study published in 2008 showed that ADAMTS-1, -4 and -5 were expressed in THP-1 monocytes [10]. In addition, after stimulation of the cells with PMA for 24 h ADAMTS-4 expression increased whereas that of ADAMTS-1 and -5 remained unchanged [10]. These findings differ slightly to those obtained during our investigations. A potential explanation for this inconsistency is that slightly different protocols were used for differentiation of THP-1 cells between the studies. Our study used a system that is employed by the majority of the researchers in the field and involves continuous treatment with PMA. On the other hand, the previous study tried to eliminate the direct effect of PMA on ADAMTS expression. They used conditioned media from already differentiated THP-1 cells for differentiation, or incubated the cells with PMA for 24 h and then removed the PMA for 24 h before commencement of experiments [10].

TGF-β has previously been shown to have a protective role during atherosclerosis [19]. Studies on human and mouse plaques have suggested a plaque-stabilising role for TGF-β, the cytokine acts to lower pro-inflammatory cytokine production, reduce MMP actions and increase collagen synthesis [44–46]. The attenuation of ADAMTS-4 expression by TGF-β is consistent with an anti-atherogenic plaque-stabilising role for TGF-β within atherosclerosis and backs up findings from a previous publication [16]. However, the increased expression of ADAMTS-1 and -5 suggest that these proteases have gene specific regulatory roles within atherosclerotic plaques. The gene specific differences in expression could also be down to the slight pleiotropic regulatory behaviour of TGF-β during atherosclerosis [16,19].

IFN-γ was shown to attenuate ADAMTS-1 expression, but it had no effect on the expression of ADAMTS-4 or -5 (Figs. 4–6). The differences observed when comparing findings to a previous publication (i.e. induced expression of ADAMTS-4; decreased levels of ADAMTS-1 and no effect on ADAMTS-5) [10] could be because of a slightly different protocol for differentiation of THP-1 cells, as detailed above. However, the differences cannot be because of the concentration of IFN-γ used as similar findings were obtained when dose-response experiments were carried out (Fig. 5). The previous study only used differentiated THP-1 cells to study the regulation of ADAMTS expression by IFN-γ [10] whereas we have extended the analysis to HMDM (Fig. 6), where the potential off target effects of PMA were eliminated, and the findings remained the same as THP-1 macrophages. IFN-γ has a pro-inflammatory role within atherosclerosis and acts to de-stabilise the plaque via increased MMP production and reduced collagen synthesis [17,47,48]. The results obtained here are not fully consistent with this pro-inflammatory role of IFN-γ. Previously, ADAMTS-1 has been hypothesised to accelerate plaque progression [11], yet the expression in macrophages was reduced by IFN-γ. The findings potentially highlight the sometimes pleiotropic actions of IFN-γ during inflammation and atherosclerosis [17].

Members of the TNF receptor superfamily have previously been implicated in the stimulation of MMP expression [21]. DR3 is the receptor for TL1A and activation of this receptor has been implicated in the induction of MMP-1, -9 and -13 from THP-1 cells in the presence of IFN-γ [21,49]. These findings, taken with the data obtained in this investigation, indicate that DR3 and its ligand, TL1A, have differential actions on different proteases that could influence atherosclerotic plaque stability. TL1A has been shown to have a weaker pro-atherogenic effect when acting on its own whereas co-stimulation with IFN-γ has been shown to increase its pro-atherogenic actions [21]. However, this was not the case with ADAMTS-1, -4 and -5 expression as the response obtained when TL1A and IFN-γ were together was similar to that seen with IFN-γ alone (Supplementary Fig. 3).

IL-17A and its roles during atherosclerosis are controversial [50,51]. IL-17A is a relatively weak modulator of gene expression; it could however work in combination with other cytokines to produce regulatory effects [52]. This could explain the variability in some of the *in vivo* data that has been obtained during previous studies [23,51–55]. Our studies show that IL-17A alone has no effect on the expression of ADAMTS-1, -4 or -5. We have of course analysed the action of only IL-17A so other members, particularly IL-17E and IL-17F, could play a role in regulating atherosclerotic plaque stability as they activate a range of target receptors and signalling pathways and are present within atherosclerotic plaques [56,57].

A major novel finding from this study was that when TL1A and IL-17A were added together, a synergistic response was observed that resulted in the increased expression of ADAMTS-1, -4 and -5 in differentiated THP-1 cells and HMDM (Fig. 8). This is an important observation because the understanding of how cytokines interact during different disease processes is key in the detailed delineation of the mechanistic actions of inflammatory mediators [43]. The action of the ADAMTS proteases is largely associated with pro-atherogenic endpoints [4]. In one previous study, ADAMTS-4 and -8 expression was shown to be up regulated in human atherosclerotic plaques and their expression from differentiated THP-1 cells was increased by stimulation with the pro-inflammatory cytokine, TNF-α [10]. In addition, ADAMTS-4 expression was attenuated by stimulation with the anti-atherogenic cytokine TGF-β [16]. Furthermore, the action of ADAMTS-7 during neointima formation showed that VSMC migration was dependent on the protease [58]. The results on the action of TL1A and IL-17A are consistent with the pro-inflammatory action of the proteases because both cytokines are largely considered pro-atherogenic [21,54].

## 5 Conclusion

We have demonstrated that the expression of ADAMTS-1, -4 and -5 is induced during the differentiation of monocytes into macrophages. The classical cytokines IFN-γ and TGF-β have a differential effect on the expression of these three members. On the other hand, the more recently identified cytokines TL1A and IL-17A alone have no effect on the expression of these three members but induce their levels synergistically when present together. The studies provide novel insight into the regulation of these important proteases by key cytokines implicated in atherosclerosis.
