# Supplementary material for: Regulation of ADAMTS-1, -4 and -5 expression in human macrophages: Differential regulation by key cytokines implicated in atherosclerosis and novel synergism between TL1A and IL-17
Source: Cytokine. 2013 Oct;64(1):234–42. doi: 10.1016/j.cyto.2013.06.315 (PMC3779352; doi:10.1016/j.cyto.2013.06.315)
Supplement: Supplementary data 1 — Supplementary Table and Figures [file mmc1.pdf]

Supplementary Table I

Gene	Primer Sequences (5'-3')
ADAMTS-1	F – GCACTGCAAGGCGTAGGAC<br>R – AAGCATGGTTTCCACATAGCG
ADAMTS-4	F – GGGATAGTGACCACATTGTT<br>R – AGGCACTGGGCTACTACTAT
ADAMTS-5	F – CACTGTGGCTCACGAAATCG<br>R – CGCTTATCTTCTGTGGAACCAAA
apoE	F – TTCCTGGCAGGATGCCAGGC<br>R – GGTCAGTTGTTCCTCCAGTTC
GAPDH	F – GAAGGTGAAGGTCGGAGTC<br>R – GAAGATGGTGATGGGATTTC
LPL	F – GAGATTTCTCTGTATGGCACC<br>R - CTGCAAATGAGACACTTTCTC

Supplementary Figure 1

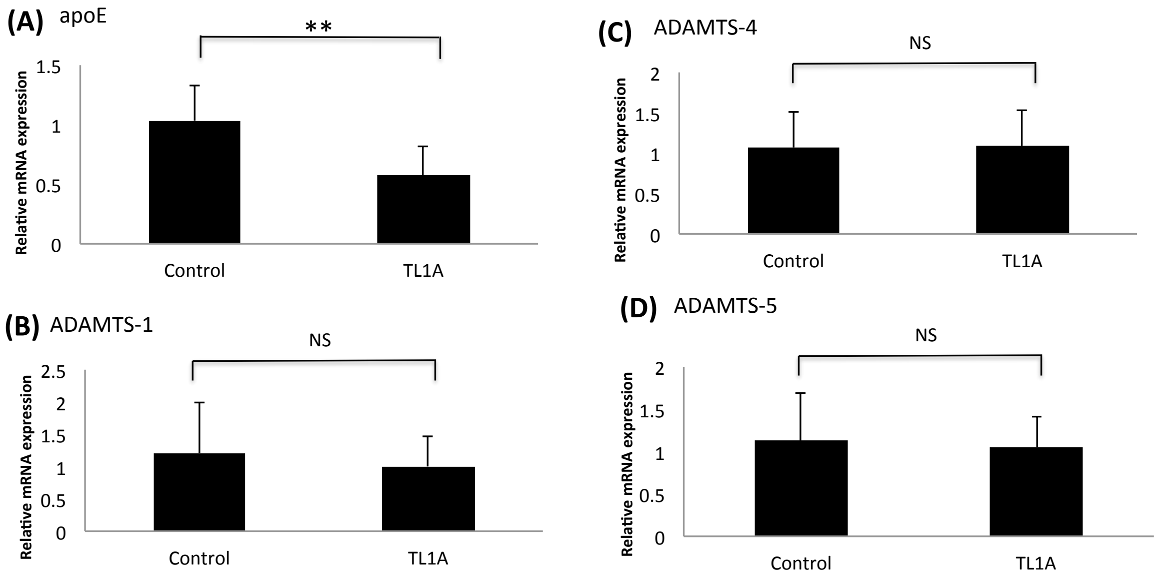

THP-1 macrophages were treated for 24h with vehicle (Control) or 100ng/ml TL1A. Total cellular RNA was isolated and subjected to RT-qPCR using primers against (A) apoE, (B) ADAMTS-1, (C) ADAMTS-4 and (D) ADAMTS-5. The mRNA expression levels were calculated using the comparative Ct method and normalised to GAPDH mRNA levels with those in control, vehicle-treated cells given an arbitrary value of 1. Data represent the mean ± SD of 3 independent experiments. Statistical analysis was performed using Student's t test (**, P<0.01; NS, not significant).

Supplementary Figure 2

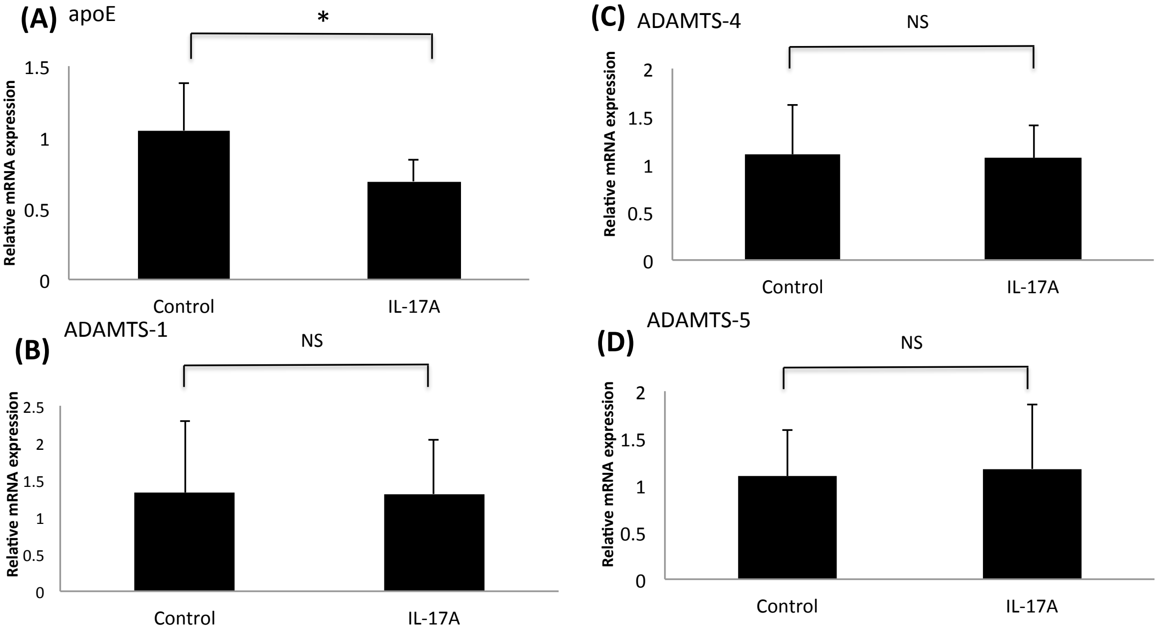

THP-1 macrophages were treated for 24h with vehicle (Control) or 100ng/ml IL-17A. Total cellular RNA was isolated and subjected to RT-qPCR using primers against (A) apoE, (B) ADAMTS-1, (C) ADAMTS-4 and (D) ADAMTS-5. The mRNA expression levels were calculated using the comparative Ct method and normalised to GAPDH mRNA levels with those in control, vehicle-treated cells given an arbitrary value of 1. Data represent the mean ± SD of 3 independent experiments. Statistical analysis was performed using Student's t test (*, P<0.05; NS, not significant).

Supplementary Figure 3

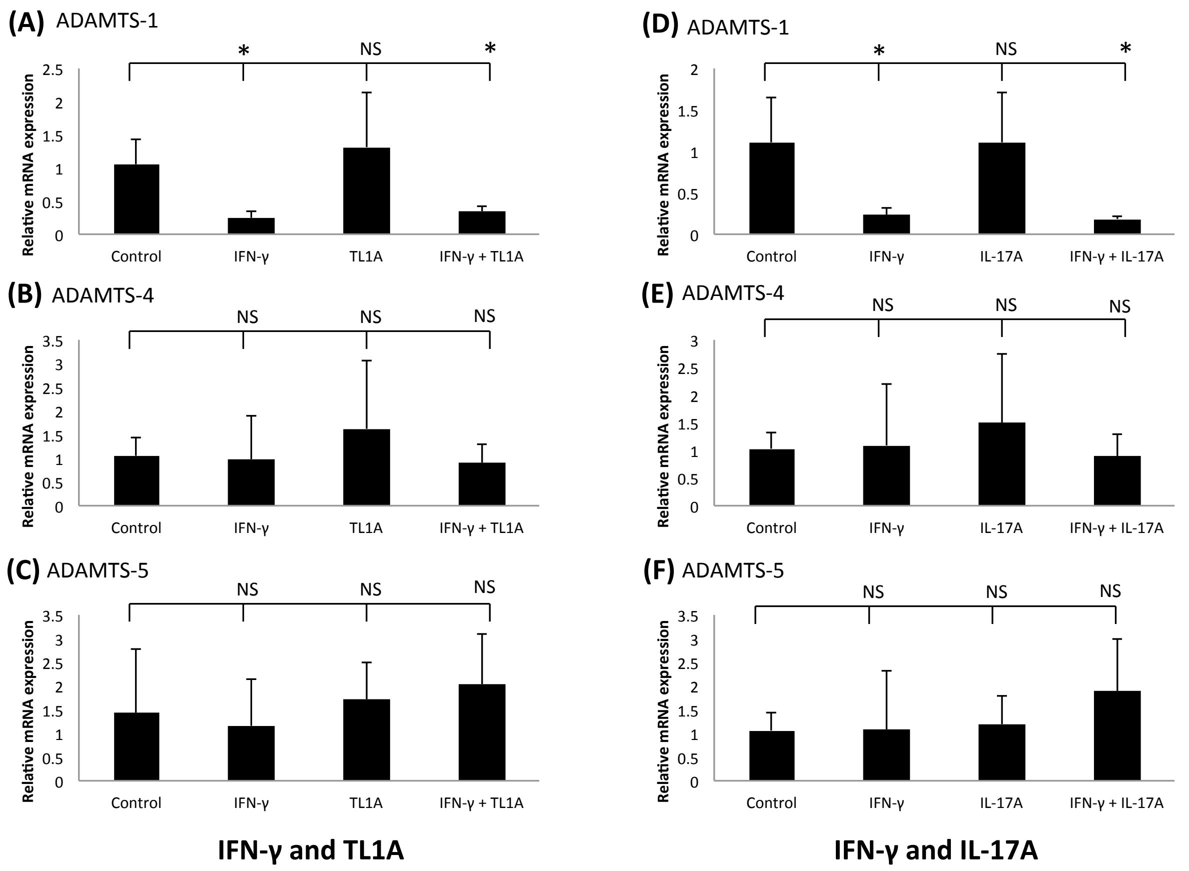

THP-1 macrophages were treated for 24h with either vehicle (Control) or 1000U/ml IFN-γ or 100ng/ml TL-1A or 100ng/ml IL-17A alone or together, as indicated. Total cellular RNA was then isolated and subjected to RT-qPCR using primers against (A, D) ADAMTS-1, (B, E) ADAMTS-4 and (C, F) ADAMTS-5. The mRNA expression levels were calculated using comparative Ct method and normalised to GAPDH mRNA levels with samples from cells treated with vehicle given an arbitrary value of 1. Data represent the mean ± SD of 3 independent experiments. Statistical analysis was performed using one-way ANOVA (*, $P<0.05$; NS, not significant).
